# The effect of “moderately restricted carbohydrate” diet on gut microbiota composition and metabolic parameters in women with metabolic syndrome: a study protocol for a randomized controlled trial

**DOI:** 10.1186/s13063-022-06922-5

**Published:** 2022-11-26

**Authors:** Seyed Mohammad Mousavi, Hanieh-Sadat Ejtahed, Hanieh Malmir, Seyed Davar Siadat, Shirin Hasani-Ranjbar, Bagher Larijani, Ahmad Esmaillzadeh

**Affiliations:** 1 Department of Community Nutrition, School of Nutritional Sciences and Dietetics, Tehran University of Medical Sciences, Tehran, Iran; 2 Obesity and Eating Habits Research Center, Endocrinology and Metabolism Clinical Sciences Institute, Tehran University of Medical Sciences, Tehran, Iran; 3 Microbiota Research Group, Endocrinology and Metabolism Research Center, Endocrinology and Metabolism Clinical Sciences Institute, Tehran University of Medical Sciences, Tehran, Iran; 4 Department of Mycobacteriology and Pulmonary Research, Microbiology Research Center, Pasteur Institute of Iran, Tehran, Iran; 5 Endocrinology and Metabolism Research Center, Endocrinology and Metabolism Clinical Sciences Institute, Tehran University of Medical Sciences, Tehran, Iran; 6 Food Security Research Center, Department of Community Nutrition, Isfahan University of Medical Sciences, Isfahan, Iran

**Keywords:** Carbohydrate-restricted diet, Metabolic syndrome, Gut microbiota, Randomized control trial

## Abstract

**Background:**

Metabolic syndrome (MetS) is a group of risk factors that increase the risk of death and a variety of chronic diseases. Recent studies have indicated that the imbalance of gut microbiota might contribute to development and progression of metabolic syndrome. Carbohydrate restriction in the diet has been proven to be one of the most effective methods in the management of metabolic syndrome, even in the absence of weight loss. However, no study has examined the effects of a carbohydrate-restricted diet on gut microbiota composition in metabolic syndrome patients. Thus, we will examine the effects of a “moderately restricted carbohydrate (MRC)” diet on gut microbiota, insulin resistance, and components of MetS among Iranian women. In addition, the stability of changes in dependent variables, including gut microbiota, will also be assessed.

**Methods:**

This is a parallel randomized clinical trial in which 70 overweight or obese women aged 20–50 years with MetS will be randomly assigned to receive either MRC diet (42–45% carbohydrate, 35–40% fats) or a normal weight loss (NWL) diet (52–55% carbohydrate, 25–30% fats) for 3 months. Protein accounted for 15–17% of total energy in both diets. The quantity of gut microbiota including Firmicutes, Bacteroidetes, *Bifidobacteria*, *Lactobacillus*, *Clostridium*, *Prevotella*, *Bacteroidetes*, and *Akkermansia muciniphila*, as well as anthropometric, blood pressure, and metabolic parameters will be measured at study baseline and the end of trail. At the end of this phase, all participants will be placed on a weight maintenance diet for an additional 6 months. After following up study subjects in this duration, all dependent variables will be examined again to assess their stability over this period.

**Discussion:**

To the best of our knowledge, this is the first randomized controlled trial investigating the effects of a moderately restricted carbohydrate diet on gut microbiota composition and several metabolic parameters during the weight loss and maintenance phases in women with MetS.

**Trial registration:**

Iranian Registry of Clinical Trials (www.irct.ir, IRCT20210307050621N1). Registered on May 31, 2021.

**Supplementary Information:**

The online version contains supplementary material available at 10.1186/s13063-022-06922-5.

## Background

Metabolic syndrome (MetS) is a group of metabolic abnormalities that include hyperglycemia, hypertension, elevated triglyceride levels, low HDL cholesterol levels, and abdominal obesity. Each of these abnormalities contribute to an increased risk of developing a variety of non-communicable diseases and mortality [1, 2]. The prevalence of metabolic syndrome is increasing as obesity rates rise globally; by 2030, it is predicted that near 50% of the population will be obese, with half of them suffering from severe obesity [3, 4]. In line with obesity trends, approximately 35% of US adults have MetS [5, 6]. In Iran, the prevalence of metabolic syndrome is estimated to be around 31% in adult population [7]. In addition, Iranian women are more likely than women in western nations to suffer from this syndrome [8].

Over the last decade, evidence inspired by gut microbiota studies has revealed the possibilities and challenges of novel metabolic syndrome prevention and treatment strategies [9]. The human microbiota consists of over 100 trillion symbiotic microorganisms that live on and within humans, and it contains roughly 150 times the number of genes found in the human genome [10, 11]. There has been a long history of mutually beneficial interactions between the gut microbiota and the mammalian host, and this has led to the development of an intricately intertwined symbiotic relationship that affects a wide range of physiological functions [12]. To understand the rise in the metabolic syndrome prevalence, one must consider the rapid changes in our environment, which have a negative impact on factors such as sedentary behavior and dietary habits [13]. Apart from several other factors including genetics, diet is considered to be the most influential factor affecting the alterations in the diversity and composition of gut microbiota [14, 15]. These factors, by mediating alteration in the gut microbiome composition, may have an effect on the immune and metabolic systems, thereby influencing the risk of MetS [16]. The gut microbiota composition of patients with MetS differs from that of healthy individuals, which has been linked to changes in intestinal permeability, inflammation, obesity, and insulin resistance [17, 18]. Dysbiosis, defined as an imbalance of gut microbiota, is implicated in the development of the metabolic syndrome [19]. Dysbiosis results in a loss of microbial diversity, the eradication of beneficial microorganisms, and the spread of potentially pathogenic microorganisms [20]. The role of gut dysbiosis in the development of immune system dysfunction, cardiovascular disease, obesity, diabetes, metabolic syndrome, asthma, and inflammatory bowel disease has been examined extensively in many studies [19, 21].

Diet plays a crucial role in the development and composition of the gut microbiota. Various dietary factors influence the gut microbiota. Among others, the quantity and balance of macronutrients (carbohydrates, protein, and fat) have attracted a particular attention [22]. Dong et al. found that a calorie-restricted high protein diet had beneficial effects on microbial diversity and alteration in *Akkermansia spp*. and *Bifidobacterium spp*., as well as the loss of *Prevotella spp*., when compared to baseline [23]. On the other hand, a high-carbohydrate diet has been linked to dysbiosis and an increase in anaerobic pathogen species, most notably *Bacteroides* [24]. No specific diet has been recommended to manage patients with MetS so far. Several studies have demonstrated that the Mediterranean and DASH diets are effective at reducing insulin resistance and other components of metabolic syndrome [25, 26]. However, none of these diets have focused on the dietary macronutrient composition. Despite the worldwide recommendation on reduced fat intake, the prevalence of this syndrome has increased [27]. Therefore, it seems that the advice on low fat diets, in spite of their beneficial effects on some components of this syndrome, might not be an appropriate choice for managing metabolic syndrome [28]. High-protein diets have also been linked to kidney dysfunction, acidosis, and insulin resistance [29]. However, dietary carbohydrate restriction might be a suitable choice because earlier studies have shown that consumption of this dietary pattern has been resulted in improved components of MetS, even in the absence of weight loss [30]. Furthermore, low-carbohydrate diets outperform low-fat diets in terms of weight loss and adipose tissue reduction [31, 32]. This is particularly relevant in Middle Eastern countries, where people get more than 60% of energy from carbohydrates, in particular from refined sources [33]. High carbohydrate intake has also been blamed as one of the primary causes of the high prevalence of metabolic syndrome in Iranians [34]. In this regard, Rajaie et al. conducted a randomized crossover clinical trial in which they applied a carbohydrate-restricted diet by substituting dietary fats; this regimen contained 43–47% carbohydrate and 36–40% dietary fat. After 3 months of follow-up, a moderate-restricted carbohydrate diet reduced blood pressure, waist and hip circumferences, and the prevalence of the metabolic syndrome [35]. To the best of our knowledge, no earlier study has examined the effect of such dietary pattern on the gut microbiome composition in patients with metabolic syndrome. Understanding the diversity and composition of gut microbiome will aid researchers in developing novel MetS prevention and treatment strategies. As a result, the aim of this study is to (i) determine the effect of a moderately restricted carbohydrate diet on gut microbiota composition and metabolic parameters in women with metabolic syndrome and (ii) determine whether these potential changes in the gut microbiota will be sustained during a subsequent six-month weight maintenance phase.

## Material and methods

The present study is a parallel two-phase randomized controlled clinical trial that will be conducted at Shariati Hospital in Tehran, Iran. All participants must complete and sign an informed written consent form prior to completing their registration (Supplementary file, section A). Ethical approval has been obtained by the Bioethics Committee of Tehran University of Medical Sciences (No. IR.TUMS.MEDICINE.REC.1400.200).

### Study population

The current single-blind randomized controlled trial is a two-phase community-based research project. Participants will be recruited through a popular online advertising program rather than a health care center or hospital. This will be done to better reflect population diversity and produce more generalizable results across populations.

### Inclusion criteria

This study will be conducted on adult women with metabolic syndrome who have body mass index (BMI) greater than or equal to 25 and aged 20–50 years. MetS was defined as having three of the five conditions listed in the Adult Treatment Panel III guidelines from the National Cholesterol Education Program (NCEP-ATP III) [36]: (1) waist circumference of more than 88 cm (35 inches), (2) serum triglyceride (TG) level greater than 150 mg/dL, (3) serum high-density lipoprotein cholesterol (HDL-C) level of less than 50 mg/dL, (4) blood pressure greater than 130/85 mm Hg, and (5) fasting plasma glucose (FPG) levels greater than 100 mg/dL. Patients who reside in Tehran will be included in the study to minimize loss to follow-up. This study will only include women because it has been demonstrated that women are more likely than men to maintain dietary changes and adhere to the prescribed diet regimen after participating in a dietary intervention trial [35, 37].

### Exclusion criteria

**Fig. 1:**
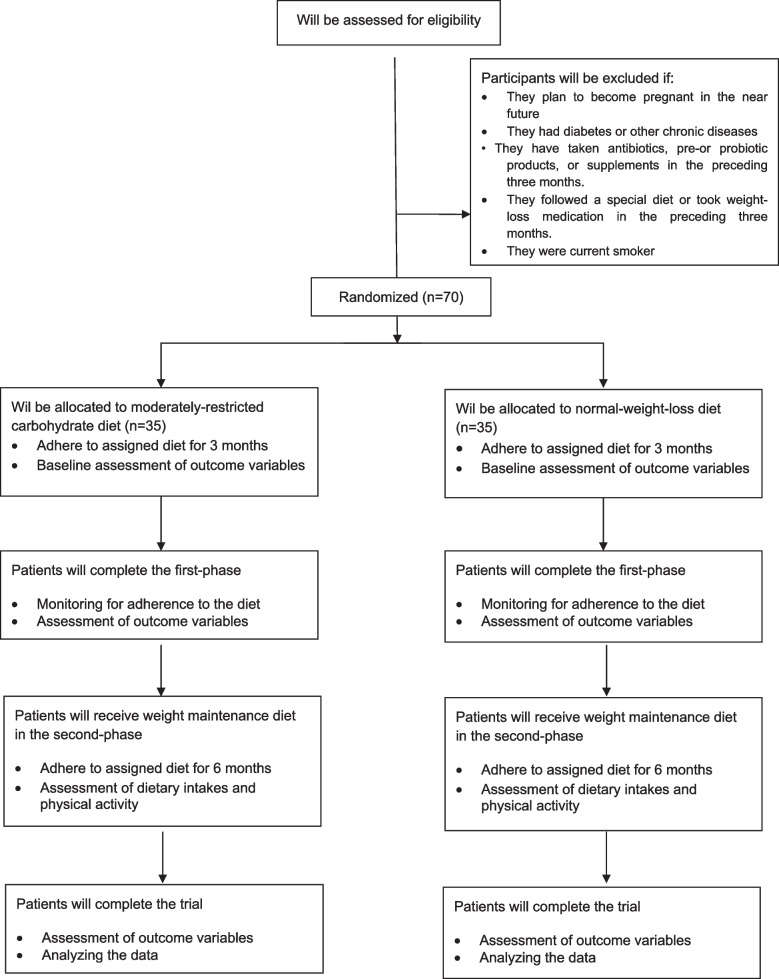
Flow chart of the study process

Patients who meet the following criteria will be excluded from the study: (1) pregnancy, breastfeeding, or planning to become pregnant in the near future; (2) being current smokers or using other tobacco such as hookah; (3) evidence of some pathologic conditions such as liver, kidney, thyroid, and gastrointestinal tract diseases as well as suffering from diabetes, rheumatoid arthritis, lupus, severe infection, and trauma; (4) weight loss history surgery; (5) having a severe allergy to specific foods; (6) have been on a special diet or taken weight loss medication for three months prior to the study; and (7) consumed antibiotics, pre- or probiotic products or supplements 3 months prior to enrollment. We will also exclude postmenopausal women, participants who are unwilling to continue the intervention and have poor adherence to the prescribed (their dietary records show that their intake deviates more than 300 kcal from the prescribed amount, or dietary records show that the recommended macronutrient composition is not followed), and those who become infected with COVID-19 during the intervention. Figure 1 presents a schematic representation of the overall research plan.

### Sample size calculation

Using a type I error of 5% (*α* = 0.05) and a type II error of 20% (*β* = 0.20, power = 80%), and serum HDL-C concentrations as the key variable, the sample size in this study was calculated using the following formula, which has been proposed for parallel clinical trials:

$$n=\frac{2\left[{\left({\textrm{Z}}_{1-\frac{\alpha }{2}}+{\textrm{Z}}_{1-\beta}\right)}^2\times {\upsigma}^2\right]}{{\textrm{d}}^2}$$

*n* = sample size for each group.

*σ* = The variance (SD) for mean HDL-C concentrations was as 3.5, which was calculated using the average SDs reported for serum HDL-C in Rajaie et al. study [35].

*d* = The minimal clinically important difference for HDL-C was set at 2.5, based on previous research [38].

Overall, using this formula and assuming a 10% drop-out rate in each group, we will require a sample size of 35 subjects in each group.

### Study design

**Fig. 2:**
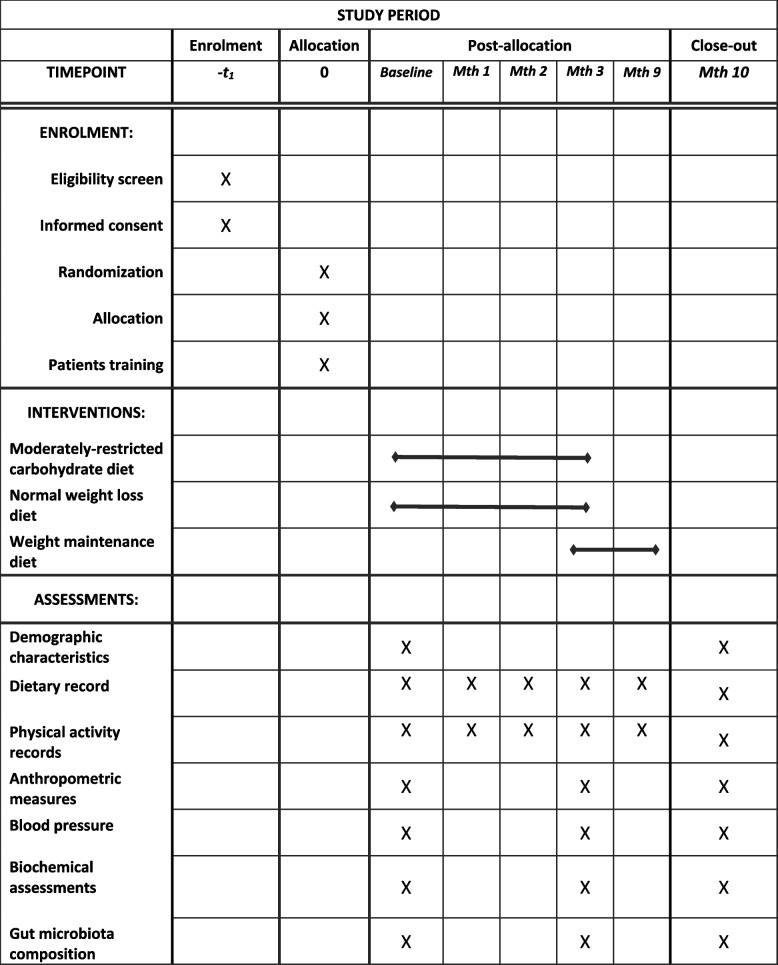
Enrollment, interventions, and assessments at various points in time. The “X” denotes what has been accomplished during a specified time period. Mth, month

A total of 100 women with metabolic syndrome will be screened according to the aforementioned inclusion criteria in the initial phase. After recruiting participants from online advertisements and social media, seventy women who meet the inclusion criteria will be included in the entire project. Patients will be enrolled using a stratified block randomization procedure with a block size of 4. Participants will be randomly assigned to the intervention or control groups based on stratification by age (20–30, 30–40, and 40–50 years) and BMI (25–29.9, 30–34.9, and > 35 kg/m^2^). First, we will enroll a woman in a stratum that meets the inclusion criteria. Following that, the second person matched based on these variables with the first one will be placed in the same stratum. Consequently, two participants with similar characteristics (for the aforementioned variables) would be in the same stratum (Supplementary file, section B). Finally, using a random allocation software, two subjects from the same strata will be randomly assigned to either moderately restricted carbohydrate diet (MRC) or normal weight loss (NWL) diet groups. The principal investigator (SMM) will enroll eligible women. All participants will sign a consent form before inclusion in the study, in which information about the prescribed diets, duration of the study, and contact information for the research team are provided. Study participants will be informed that the services given to them will be free of charge and that they can withdraw from the study at any time throughout the study. Each person will receive a booklet, including a pre-designed dietary plan containing a series of menus and recipes for breakfast, lunch, dinner, and snacks according to the prescribed diet. In the current trial, outcome assessors will be blinded to the intervention allocation. The study protocol for this clinical trial was registered with the Iranian Registry of Clinical Trials in advance (IRCT20210307050621N1). The flow diagram of the research process is presented in Fig. 2.

### Intervention

Participants will be randomly assigned into one of the two above-mentioned groups. During an hour-long discussion, each participant will be instructed on either the MRC diet or NWL diet. All participants will receive a calorie restricted diet based on their status of weight and BMI (300–500 kcal less than participant’s energy requirement). We will compute calorie requirements based on resting energy expenditure (using the Harris-Benedict equation) and physical activity levels. The main distinction between these two diets will be the proportion of calories derived from fats and carbohydrates. The MRC diet will be defined as 42–45% of energy from carbohydrates and 35–40% from fats, while the NWL diet will be defined as 52–55% of total calories from carbohydrates and 25–30% from fats. In fact, 10% of energy from carbohydrates in the MRC diet will be substituted with non-hydrogenated vegetable oils. Dietary protein intakes will be accounted for 15–17% of total energy across both dietary groups. The first phase of the study in which subjects will receive the aforementioned diets will last for 3 months. Then participants will enter the second phase, in which all participants will be followed for another 6 months on a weight-maintenance (WM) diet containing a normal proportion of macronutrients, including 52–55% carbohydrates, 27–30% fat, and 15–17% protein. The main objective of this phase is to determine whether the changes caused by the intervention will be maintained after six months. In addition, it will be determined if changes in gut microbiota persist after 6 months of a weight-maintenance diet and to what extent these changes might contribute to weight maintenance and the stabilization of metabolic variables after 6 months.

### Compliance

To assess how well participants adhere to their diets, their dietary intakes will be recorded once every 2 weeks in the first phase and every 4 weeks in the second phase by telephone interview. A sample of dietary recall format is provided in Supplementary file, section C. In total, participants are going to complete 12 dietary records (six in the first phase and six in the second phase). Participants will be asked to report their dietary intakes using household measures in order to complete the dietary recalls. Then, we will convert household measures to grams using the booklets provided. Participants’ total dietary intakes will be assessed using the average of all dietary recalls taken over the duration of the intervention. Total energy intake, macronutrients, and micronutrients will be computed from the dietary recalls using Nutritionist-IV software (First Databank, San Bruno, CA, USA) modified for Iranian dishes. We will also send text messages and make phone calls to participants throughout the study to increase compliance and prevent forgetfulness of diet principles.

### Intervention safety

There have been no serious side effects associated with moderate carbohydrate restriction diets. To investigate any potential side effects, we will ask participants to report any changes during the study period concurrent with their dietary assessment via phone interview.

### Assessment of variables

A standard questionnaire will be used to collect data on participants’ age, ethnic origin, marital status, educational level, disease history, family history of disease, medication use, and history of COVID-19 and type of vaccination. We will also measure primary outcomes including serum lipid profile concentrations, fecal bacterial load, and glucose levels, as well as secondary outcomes including anthropometric measurements and blood pressure at the beginning of the study, end of the phase I (3 months), and end of the phase II (6 months).

### Biochemical assessments

A blood sample will be taken from each patient’s venous blood at the study baseline, end of the first phase (after 3 months), and end of the second phase of the study (after 9 months) following 12 h of fasting. We will use a portion of the blood sample to evaluate glucose levels and lipid profile. The remaining serum will be kept at – 80 °C until it is further analyzed for insulin levels. Plasma glucose and lipid profile concentrations (TC, HDL, LDL, and TG) will be determined via an enzymatic colorimetric method using commercial kits. Finally, the serum insulin concentrations will be examined using electrochemiluminescence (ECL) method. We also will determine insulin resistance (HOMA-IR) and quantitative insulin-sensitivity check index (QUICKI) formulas.

### Stool samples and gut microbiota assessment

A 10-g stool sample will be collected from each participant at study baseline, 3 months after intervention, and 6 months after the end of intervention using a stool specimen collection kit that will be brought to the clinic in ice packs within 4 h. Until fecal microbial analysis, stool samples will be frozen at – 80 °C in the laboratory. To minimize changes in the quantity of fecal bacteria, we will collect stool samples in one of three ways: first, we will invite patients to visit the laboratory on the day of fecal sampling. The lab will be equipped with all of the necessary equipment to collect fecal samples. The second method requires the patient to transport the sample to the laboratory within 4 h of their sampling. Finally, if the patient is unable to complete any of the prior approaches, they should contact the study researchers, who will collect their sample at their home and transfer it within 4 h to the lab.

DNA extraction from 200 mg of frozen stool samples will be done using a FavorPrep Stool DNA Isolation Mini Kit (Favorgen Biotech Corp., Taiwan) according to the manufacturer’s instructions. The extracted bacterial DNA purity and concentration will be determined by Nanodrop spectrophotometer (Thermo Scientific NanoDrop, USA). All the extracted DNAs will be stored at − 20 °C freezer until further analysis. Additionally, the quantity of fecal bacteria, including Firmicutes, Bacteroidetes, *Bifidobacteria*, *Lactobacillus*, *Clostridium*, *Prevotella*, *Bacteroidetes*, and *Akkermansia muciniphila*, will be determined using a quantitative real-time polymerase chain reaction (qRT-PCR) in duplicate. We selected the taxa based on their association with metabolic syndrome and their ability to balance the gut microbiota [39, 40]. Specific primers targeting the bacterial 16S rRNA genes blasted in NCBI will be used in the current study. Each reaction mixture is composed of SYBR Premix Ex Taq II (Takara, China), the specific forward and reverse primers, and a DNA template. The thermal cycling conditions will be as follows: an initial DNA denaturation step at 95 °C for 1 min, 40 cycles of denaturation at 95 °C for 5 s, primer annealing at 55 °C for 30 s, and extension at 72 °C for 30 s. Finally, in order to confirm the specificity of the amplification products, melting curve analysis will be performed by slowly cooling the PCRs from 95 °C to 60 °C.

### Anthropometric measures

Weight will be determined with the minimum clothing without shoes using a weighing calibrated scale (Seca, Hamburg, Germany) to the nearest 100 g. Standard stadiometer will be used to measure barefoot standing height to the nearest 0.5 cm. Measurement of the waist and hip circumferences will be done to the nearest 0.5 cm using a strip tape measure over light clothing without applying pressure to the body surface. The waist and hip circumferences will be defined as the circumference around the belly button and the diameter of the hip’s largest point. The body mass index (BMI) will be calculated by dividing weight in kilograms by the height in meters squared.

### Blood pressure assessment

After at least 5-min resting, measurements of systolic and diastolic blood pressure will be done twice with 15-min intervals in the right arm using a mercury barometer calibrated by the National Institute of Standards and Industrial Research. The average of two blood pressure readings will be used to determine an individual’s blood pressure.

### Physical activity assessment

We will determine the level of physical activity of participants based on their once in a month physical activity recording (including working and non-working days) (Supplementary file, section D). Physical activity levels will be classified as low, moderate, or high and expressed in metabolic equivalents per week (MET-min/week).

### Confidentiality

The information about participants will be stored anonymously according to TUMS rules. During laboratory tests and in all records, participants will only be able to be identified by their ID number so as to assure their privacy and confidentiality. In a secure database, we will store participants’ identifying information separately from the research data collected during the study. Moreover, all specimens will be stored in the TUMS biochemistry laboratory. All study data will be kept strictly confidential on password-protected computers in the research team’s office, which will be accessible only by the research team. The corresponding author will provide anonymized data to other researchers in order for them to conduct secondary studies after the trial has been completed on reasonable request.

### Data management and monitoring

Principal study coordinators will be SMM and BL, who have designed the study and will be responsible for project oversight. In fact, the Trial Steering Committee (TSC) is comprised of AE and BL as key members, the study physician (SHR), the study assistant and study advisor (SDS), the statistician and study advisor (HSE), and the principal investigator (SMM). Monthly meetings of this committee will be held to assess performance and progress, overcome technical and financial challenges, and develop plans to meet project deadlines. A separate data monitoring committee is not required due to the low-risk nature of the intervention. The laboratory of the Endocrine and Metabolism Research Institute at Tehran University of Medical Sciences serves as the coordinating canter, where blood sample, serum collection, storage, and all laboratory testing are conducted. Main investigator (SMM) is responsible for coordinating visits for identifying potential participants and obtaining consent. There will be a Data Management Team comprising the principal investigator (SMM) and the project supervisors (AE and BL).

### Statistical analysis

All statistical analyses will be carried out using the SPSS software version 25 (SPSS Inc., Chicago, IL, USA). For addressing non-adherence, the intention-to-treat (ITT) analysis will be applied [41]. Moreover, we will apply the last observation carried forward method (LOCF) to handle missing values [42]. In order to determine the normal distribution of variables, we will use the Kolmogorov-Smirnov test, histogram, and Q-Q plot. We will log transform (ln) the data, if necessary, to achieve normal distribution. Data will be presented as mean ± standard deviation (SD) for continuous variables and as percentages for categorical variables. Chi-square test and independent samples *t*-test will be used for comparing continuous and categorical variables between the two groups, respectively. We also will use the paired-sample *t* test for within-group comparisons. Repeated measures analysis of variance will be employed to determine the impact of the intervention on the outcome variables. These analyses will examine the effects of the intervention, time, and the interaction between time and intervention. Any potential differences in baseline levels of outcome variables, dietary intakes, and physical activity will be controlled in these analyses. Statistical significance will be defined as *P* values less than 0.05.

## Discussion

Metabolic syndrome is currently regarded as one of the most important leading causes of non-communicable diseases, and its global prevalence is rapidly increasing, imposing a considerable burden on the health care system [43]. Obesity caused by dietary changes is the leading cause of metabolic disorders, which might eventually lead to diabetes and heart disease [44]. Carbohydrate-restricted diets, despite lacking a precise definition, have attracted considerable attention in recent decades due to their widespread use for weight loss and weight management [45]. Dysbiosis of the gut microbiome composition are currently being considered as one of the potential risk factors of MetS [46]. It has been established that approximately 100 trillion bacteria and other microbes reside in the human gut microbiome [46]. Humans and microbes have a long evolutionary history, and numerous microbes have a significant impact on human health. However, technological advancements have drastically changed environmental conditions. Many of these changes are quite significant [47]. Among these, the relationship between diet and gut microbiome composition has received more attention [48]. Recent dietary changes including a high carbohydrate intake have been linked to decreased microbiome diversity, increased inflammation, and an increased risk of metabolic disease and may have contributed to the development of MetS [18]. Since metabolic syndrome is caused by a complex interaction between the underlying causes and the complications, employing a safe and functional dietary intervention combined with a practical delivery method to halt the rapid increase in metabolic syndrome prevalence would be effective.

In this study, we hypothesize that following a moderately restricted carbohydrate diet would decrease MetS components by inducing changes in the gut microbial community. The findings of the present study may shed light on the implementation of an appropriate dietary strategy to facilitate and accelerate weight loss and improve metabolic syndrome components.

## Strengths and limitations

This is the first clinical trial examining the effect of a moderately restricted carbohydrate diet on the composition of gut microbiota and several metabolic parameters in patients with MetS. It should be noted that the intervention is low-cost and no extra charge would be imposed to patients. Stratified block randomization will be used to match patients based on a number of confounding variables that may influence the outcomes. Additionally, participants will be recruited through public announcements. Thus, all volunteers may be encouraged to adhere to dietary recommendations. Another potential strength of the study is the measurement of gut microbiota composition and biochemical parameters not only during the intervention but also after the intervention, during the maintenance phase. Dietary intakes and physical activity will be evaluated during both phases of the study as well.

There are several limitations to this study that should be considered. The major limitation of the current trial is the small number of bacterial species and biochemical parameters that will be examined. Moreover, we are concerned about gut microbiota changes during sample collection. In order to prevent this, stool samples will be collected in the laboratory and immediately frozen at − 80 °C. In addition, we strive to have all stool samples delivered to the laboratory within 4 h of collection. Nevertheless, several samples may arrive within more than four hours. Even though biomarkers could provide more accurate measures for validating dietary records, we will not use them to evaluate diet compliance due to financial constraints. This study will be conducted during the COVID-19 pandemic, which could increase the number of participants who are lost to follow-up.

## Trial status

The recruitment phase in the trial began in December 2021 and is ongoing. We anticipate the recruitment to end in June 2022. Protocol modifications will be communicated to all relevant parties, and the Iranian Clinical Trials Registry will be updated accordingly (IRCT.ir).

## Supplementary Information

**Additional file 1: A.** Consent form. **B.** Randomization procedure diagram. **C.** Food recall. **D.** Physical activity record.

## Data Availability

Any data to support the protocol can be provided upon request by corresponding author.

## Abbreviations

MetS: Metabolic syndrome
MRC: Moderately restricted carbohydrate
NWL: Normal weight loss
HDL: High-density lipoprotein
DASH: Dietary Approaches to Stop Hypertension
NCEP-ATP III: Panel III guidelines from the National Cholesterol Education Program
FPG: Fasting plasma glucose
BMI: Body mass index
WM: Weight-maintenance
ITT: Intention-to-treat
LOCF: Last observation carried forward method
SD: Standard deviation
QUICKI: Quantitative insulin sensitivity check index
HOMA-IR: Hemostatic model assessment of insulin resistance
ECL: Electrochemiluminescence
